# The Work Experience Survey – Rheumatic conditions (United Kingdom): Psychometric properties and identifying the workplace barriers of employed people with inflammatory arthritis receiving vocational rehabilitation

**DOI:** 10.1002/msc.1835

**Published:** 2023-10-25

**Authors:** Alison Hammond, Rachel O’Brien, Sarah Woodbridge, Jennifer Parker, Angela Ching

**Affiliations:** ^1^ Centre for Human Movement and Rehabilitation Research School of Health and Society University of Salford Salford Greater Manchester UK; ^2^ Occupational Therapy School of Health & Wellbeing Sheffield Hallam University Sheffield UK; ^3^ (formerly) Work Fit Work Safe University Hospitals of Derby and Burton Derby UK; ^4^ The University of Queensland Faculty of Health and Behavioural Sciences Metro North Health, Surgical Treatment and Rehabilitation Services (STARS) Hertson Queensland Australia

**Keywords:** arthritis, musculoskeletal, vocational rehabilitation, work, work assessment

## Abstract

**Objective:**

The aims were to: revise the Work Experience Survey‐Rheumatic Conditions (WES‐RC‐ UK), a work assessment listing 142 workplace barriers; investigate content validity, reliability, and concurrent validity; update the accompanying WES‐RC and WORKWELL Solutions Manuals; and investigate workplace barriers of people with inflammatory arthritis.

**Methods:**

Rheumatology therapists, following vocational rehabilitation (VR) training, assessed participants in the WORKWELL VR trial using the WES‐RC. Data were extracted from the WES‐RC to identify the frequency of workplace barriers, and from trial baseline questionnaires (e.g., Work Limitations Questionnaire‐25 (WLQ‐25). Barriers reported by ≤5 participants were considered for removal. WES‐RC content validity was assessed by linking to the International Classification of Functioning, Health, and Disability Core Set for VR (ICF‐VR). Reliability was assessed using Cronbach's α and concurrent validity by correlating the total number of workplace barriers reported with WLQ‐25 scores.

**Results:**

WES‐RCs were completed with 116 employed participants: 79% women, age 48.72 (SD 9.49) years, and 57% working full‐time. The WES‐RC was reduced to 121 barriers. Content validity was good, with 73/90 ICF‐VR items linked. Cronbach's α = 0.92, that is, suitable for individual use. Concurrent validity was moderate: WLQ‐25 (*r*_*s*_ = 0.40). The three most common barriers were Physical Job Demands (100%: e.g., mobility 99%; hand use 74%), Mental, Time, Energy, Emotional Job Demands (91%, e.g., concentration 47%, remembering 41%); Getting Ready for and Travel to Work (87%, e.g., driving 60%).

**Conclusion:**

The WES‐RC (UK) has good content validity, reliability, and concurrent validity. The wide range of barriers emphasises the need for biopsychosocial work rehabilitation.

## 1 INTRODUCTION

Reduced at‐work productivity due to ill‐health (i.e., presenteeism) is experienced by 53%–79% of working people with rheumatoid arthritis (RA) (Braakman‐Janssen et al., 2012; Kim et al., 2017). People with RA can still experience significant limitations in their work ability, even if on biological drugs (Gwinnutt et al., 2020). Within five to 10 years of symptom onset, 20%–70% prematurely stop work due to ill‐health (Verstappen, 2015). The European Alliance of Rheumatology Associations (EULAR) recommends that work‐related support for people with rheumatic and musculoskeletal diseases (RMDs) should consider all aspects of the biopsychosocial framework of health to address their work participation needs. That is, body functions and structures, work‐related activities and participation, as well as personal and work environmental contextual factors, as relevant, in the International Classification of Functioning, Disability and Health (ICF) (Boonen et al., 2023; World Health Organisation, 2001).

The Work Experience Survey‐Rheumatic Conditions (WES‐RC) is a comprehensive biopsychosocial evaluation of the workplace difficulties experienced by working people with RMDs. It is a semi‐structured interview schedule which can be conducted anywhere, either in‐person or by telephone/videocall, by healthcare professionals (HCPs) and occupational health personnel (OHP). The WES‐RC includes demographic, health, and job‐related information, as well as listing 120 potential workplace difficulties (AlHeresh et al., 2023; Allaire & Keysor, 2009). The final section of the WES‐RC consists of jointly planning with the client an individualised job retention vocational rehabilitation (JRVR) programme. The WES‐RC has been used in two JRVR trials in RMDs in the United States (US) (Allaire et al., 2003; Keysor et al., 2018).

The WES‐RC was adapted for use in RMDs, from the original WES (Roessler, 1995), through literature review and focus groups with working people with arthritis, supporting its content validity (Allaire & Keysor, 2009). Other aspects of validity and reliability have not been investigated. Whilst the WES‐RC is not an outcome measure, it is still appropriate to ensure that it has a degree of reliability and validity appropriate for individual clinical use. It is designed to evaluate the fit between a worker's abilities, their job demands, and their work environment (Roessler et al., 2017). It is therefore unlikely that people will experience the full range of difficulties, or barriers, listed, as these are influenced by the nature of the person's job and work environment, as well as their level of ability. The WES‐RC was adapted for use in the United Kingdom (UK) for a feasibility trial of JRVR in inflammatory arthritis (IA) (Hammond et al., 2017). To investigate its applicability for the UK, a panel of working people with RA and VR occupational therapists reviewed content to identify any changes needed due to differing health, social or employment‐related factors between the US and the UK. This led to additional items, particularly in Section 6 Environmental Factors and Company Policies. Additionally, a body outline was included in Section 1 to record body structures affected (Hammond et al., 2011, 2013).

As this UK adaptation occurred over 10 years ago, the WES‐RC (UK version) needs reviewing to identify if all barriers included are still appropriate and if any new items are needed. Additionally, aspects of its validity and reliability need to investigate to support its use. A WES‐RC Manual was also developed, as well as a Workwell Solutions Manual linked to barriers in the WES‐RC, which details solutions to work barriers identified. These supported therapists in using the WES‐RC, and planning and delivering JRVR in the feasibility trial (Hammond et al., 2017). These manuals were revised for a subsequent randomised controlled trial, the Workwell trial (Hammond et al., 2020), and therefore also need updating.

The WES‐RC has been used to report the frequency of workplace barriers in RMDs (AlHeresh et al., 2023) and systemic sclerosis (Poole et al., 2016). Better understanding of the work‐related barriers experienced specifically by working people with IA can assist in future planning of work support services needed to help people with IA stay working.

Using data from WES‐RCs, and accompanying treatment notes, completed during the Workwell trial, the aims of this study were to: revise the content of the UK version of the WES‐RC, investigate its content validity, reliability (internal consistency), concurrent and discriminative validity, update the accompanying WES‐RC Manual and Workwell Solutions Manual, and investigate the workplace barriers reported by working people with IA.

## 2 METHODS

### 2.1 Study design and participants

A cross‐sectional survey was conducted using data obtained from participants with IA receiving individualised JRVR in the Workwell trial. The trial protocol and JRVR intervention are described in detail elsewhere (Hammond et al., 2020, 2022). Although the WES‐RC is not an outcome measure (rather the number of work barriers experienced can be totalled if required), some of its psychometric properties need investigating to support its use in VR. The Consensus‐based Standards for the selection of health Measurement Instruments (COSMIN) checklist were followed in reporting the study (Gagnier et al., 2021).

Participants were recruited from 18 National Health Service (NHS) out‐patient rheumatology or therapy clinics in England, Wales, and Scotland. Participants were eligible if they were at least 18 years of age, diagnosed with RA, early IA, or psoriatic arthritis (PsA) diagnosed by a rheumatology consultant, in paid work for at least 15 h/week, not on sick leave, able to read and understand English, and able to attend JRVR. To ensure that participants were applicable to receive JRVR, they needed to score ≥10 on the RA‐Work Instability Scale (RA‐WIS), a measure of mismatch between a person's work abilities and their job demands. A score of ≥10 indicates medium to high risk of work disability (i.e., prematurely stopping work due to ill‐health) and the need for JRVR (Gilworth et al., 2003). Exclusion criteria were being on extended sick leave (>4 weeks; those on short‐term sick leave could be recruited when back at work), planning to retire within 12 m, and already receiving or waiting for JRVR from elsewhere (e.g., Access to Work, a UK Government‐funded work support service ((UK Government, 2023)).

Ethical approval was obtained from the West Midlands–Solihull Research Ethics Committee (18/WM/0327). All participants provided written informed consent. The recruitment occurred sequentially between March 2019 and February 2021. The Workwell trial was affected by the COVID‐19 pandemic, with recruitment and treatment paused from mid‐March 2020, and resuming across sites between mid‐July 2020 and January 2021, dependent on when each site approved re‐start. Recruitment slowed after re‐start and therefore volunteers were recruited to reach the trial's target sample size (*n* = 240). Volunteers were identified from a University Arthritis Volunteers database and the National Rheumatoid Arthritis Society (NRAS). Changes to the trial protocol due to the COVID‐19 pandemic are detailed elsewhere (Ching et al., 2022).

### 2.2 Procedures

Patients were screened at participating sites for eligibility and consented. Participants then completed a paper version of the trial baseline questionnaire at home and mailed this to the research team. Following receipt (and collection of any missing data), participants were randomised to the trial intervention or control arms. Within two working days, all participants were then mailed a written self‐help work information pack, including four work booklets with information about the impact of arthritis on work, work problems, possible solutions and their rights at work, and asked to read these. Intervention participants were also referred to the appropriate treating therapist. Within 4 weeks, therapists arranged an initial appointment at which the WES‐RC (UK version) was completed.

Treating therapists were experienced rheumatology occupational therapists who completed a Workwell trial JRVR training course at the start of the trial, including conducting the WES‐RC, identifying priority problems, planning individualised JRVR programmes using example case studies, practical workshops in JRVR solutions, and completed a mock WES‐RC interview and JRVR treatment plan, assessed by one of the course trainers. All therapists received a WES‐RC Manual, an example of a completed WES‐RC with accompanying treatment notes, and a Workwell Solutions Manual (Hammond et al., 2021).

### 2.3 Data collection

#### 2.3.1 The Work Experience Survey – Rheumatic conditions

The WES‐RC was conducted during the intervention participants' first JRVR appointment. It includes eight sections: (1) Demographic and health information, health symptoms impacting work, and work history; (2) Getting ready for and travel to/from work; (3) Workplace access; (4) Completing job activities, divided into two parts: (A) physical demands; and (B) mental, and time/energy/emotional demands; (5) Relationships with people at work; (6) Environmental factors and company policies; (7) Job, career, and home life; and (8) Problem prioritisation and solution development. During the WES‐RC interview, participants and therapists discussed each section, identifying which workplace barriers are “sometimes or always” a problem. At the end of each section, the participant identified which of these were major (i.e., “often or fairly bothersome”). From this process, in Section 8, participant and therapist together identified the three key problem areas to address in JRVR, and jointly planned solutions and the actions needed (by both participant and therapist) to address these problems, and developed action plans that each would undertake to implement these solutions. The WES‐RC can usually be completed within an hour appointment, including identifying problems, agreeing solutions and action planning. The amount of time taken will depend on a number of factors. It can take longer if the health professional has less experience conducting semi‐structured interviews, is using the WES‐RC with a client for the first time, and is still developing knowledge of work solutions. Clients with more complex problems, particularly if more stressed or distressed about their work problems or other issues, may also require more time. During the Workwell trial, the time varied between 45 min and 1.5 h.

The WES‐RCs were conducted in‐person in NHS therapy clinics, during which participants were usually able to see the paper copy of the WES‐RC as the therapist completed it. However, following trial re‐start, most appointments had to be conducted by videocall or telephone due to social distancing and infection control requirements. The chosen method was dependent on participants' preference and sites' capability to provide videocalls. All therapists were provided with a Microsoft Word version of the WES‐RC to complete, if required, if their site had switched more quickly to electronic medical records. Many continued to complete a paper WES‐RC, but during videocalls, therapists could intermittently share a copy of the WES‐RC on‐screen with participants, if wished. An online version of the Workwell Solutions Manual was also made available to therapists, via the trial's website.

Following the completion of a participant's JRVR, therapists provided a copy of the completed WES‐RC and accompanying treatment notes to the Workwell research team for analysis. Demographic characteristics, health symptoms, and workplace barriers were extracted from the WES‐RC.

#### 2.3.2 Work and health measures

To provide additional work and health status information, and to support validity testing, data were extracted from the trial baseline questionnaire. Work‐related items included employment status and job skill level (Office for National Statistics, 2010). Three work outcome measures were included, each with good validity and reliability. The Work Limitations Questionnaire‐25 (WLQ‐25) consists of 25 items in four sub‐scales of time management, physical, mental‐interpersonal and output demands (Lerner et al., 2001). The summed score (i.e., the average of the four subscales scores) was calculated (Roy et al., 2011). Other work measures were the RA‐WIS, which has cut‐points to identify those with low (0–9), medium (10–17) and high risk (18–23) of work disability (Gilworth, et al., 2003); and the Workplace Activity Limitations Scale (WALS), also with cut‐points identifying low (0–6), medium (7–13) and high (14–36) risk of work disability (Gignac et al., 2011; Hammond et al., 2023a). Additionally, information about perceptions of physical and mental job demands and stressful job were extracted (1 = not at all; 5 = a great deal).

Health items included the SF‐12v2 general health (score 1–5) question (Ware et al., 1996) and 0–10 numeric rating scales (NRS) from the RA Impact of Disease scale of pain, fatigue, functional disability, and emotional well‐being (Gossec et al., 2011).

### 2.4 Sample size

For validation studies in which correlation coefficients are calculated, a minimum of 50, and preferably larger samples (e.g., over 100) are preferred (De Vet et al., 2011). The sample size was determined by the number of participants completing the WES‐RC with a Workwell trial therapist.

### 2.5 Statistical analysis

Data were tested for normality, and analysed using frequency counts and percentages, mean (SD) or median (IQR), as applicable. To revise the WES‐RC (Sections 2–7), first the frequency (percentage) of workplace barriers reported as “sometimes or always” a problem was identified. Items reported by ≤5% of participants were either considered for removal, combined with existing items (if applicable), or used as examples in the “other” options within sections for recording barriers not listed. The “other” responses were also reviewed and, where applicable, barriers were re‐coded to listed items. Any “other” items reported by >5 participants were considered for inclusion as new items or added to existing items.

To explore content validity, the WES‐RC was linked to the ICF Core Set for VR (ICF‐VR) (Finger et al., 2012; Supplementary Table S1) using the ICF‐linking rules (Cieza et al., 2005). A limitation of the ICF is that it does not specify personal factors. Accordingly, twelve contextual personal and work environment factors, including 25 items, have been identified as important influences on worker productivity (Boonen et al., 2021) (Supplementary Table S2). These were also linked to the WES‐RC to identify if any contextual factors needed adding.

Internal consistency, a form of reliability measuring the degree of interrelatedness between items within a scale, was assessed using Cronbach's alpha (*α*). Results ≥0.80 are deemed good to excellent, with ≥0.90 consistent with individual use, and ≥0.70 with group‐level use (Evans, 1996). To investigate concurrent validity, the number of UK WES‐RC barriers reported was correlated with WLQ‐25, RA‐WIS and WALS scores, using Spearman's correlations, as data were not normally distributed. Correlations of 0.20–0.39 are considered weak, 0.40–0.59 moderate, and ≥0.60 strong (Evans, 1996). It was hypothesised that correlations would be moderate at best, because these three measures do not address the full range of items within the WES‐RC. To investigate the extent to which they do so, the WLQ‐25, RA‐WIS and WALS were also linked to the WES‐RC. Discriminative validity of the WES‐RC Sections 2–7 was investigated using Kruskal‐Wallis tests between differing levels of work instability, using the RA‐WIS and WALS cut‐points; and health status, using the SF‐12v2 General Health item (good, fair, and poor health).

The frequency of health symptoms and workplace barriers identified as “sometimes or always a problem”, and as a major problem were then investigated. The median (IQR) number of workplace barriers in each section and in total were calculated. Differences in the number of health symptoms and WES‐RC barriers reported between the three condition groups (i.e., RA, early IA and PsA), were analysed using Kruskal‐Wallis tests, and between men and women using Mann‐Whitney tests. Data were analysed using SPSS v 26 (IBM, 2019).

## 3 RESULTS

### 3.1 Participant characteristics

The WES‐RC was completed by Workwell therapists with 116 of the 124 intervention group participants, as eight either withdrew before or did not attend treatment. Of these, 102 (88%) were recruited from NHS clinics, 4 (3%) from a University Volunteer database, and 10 (9%) from an arthritis charity. Of the WES‐RC appointments, 72 (62%) were conducted in‐person (67 of these before the trial pause), 27 (23%) by videocall and 16 (14%) by telephone. For one participant, mode was not recorded by the therapist.

Demographic, health, and work factors are reported in Table 1. Around two‐thirds were diagnosed with RA, and 79% were women. Participants had moderate pain, fatigue, functional disability, and mental wellbeing scores. Two‐thirds worked full‐time, with 51 (44%) reporting their job as physically demanding, 97 (84%) mentally demanding, and 77 (66%) as stressful. Just under half (46.5%) had level 1 or 2 jobs (i.e., unskilled, and semi‐skilled jobs); and the remainder level 3 and 4 jobs (i.e., skilled, associated professional/technical, managerial, or professional jobs). There were no demographic differences between participants recruited from the NHS or volunteers (data not shown).

**TABLE 1:** Baseline participant demographics, health and employment characteristics (*n* = 116).

	Intervention (*n* = 116)
Demographic factors:	
Age (years), mean (SD)	48.72 (9.49)
Sex (female), *n* (%)	92 (79.30)
Married/living with partner, *n* (%)	82 (70.70)
Ethnicity: White/other	112 (96.55)/4 (3.45)
ISCED education level, *n* (%)	
Low; medium; high; missing	18 (15.50); 28 (24.10); 64 (55.20); 6 (5.20)
Recruited from: NHS; volunteers, *n* (%)	102 (87.93); 14 (12.07)
Health factors from trial questionnaire:	
Diagnosis, *n* (%): RA; early IA; PsA	78 (67.24); 13 (11.20); 25 (21.55)
Time since diagnosis (years), median (IQR)	4.00 (2.00–13.00)
Medication regimen, *n* (%) (no. DMARDS)	
0; 1; ≥2; biologic/biosimilars (+/− DMARD)	7 (6.00); 41 (35.34); 25 (21.55); 39 (33.62)
Missing	1
RAID: Pain (0–10)	6.00 (4.00–7.00)
RAID: Fatigue (0–10)	7.00 (6.00–8.00)
RAID: Functional disability (0–10)	6.00 (4.00–7.00)
RAID: Emotional wellbeing (0–10)	6.00 (4.00–7.00)
SF‐12v2: General health (1–5), median (IQR)	4.00 (3.00–4.00)
Work‐related factors from trial questionnaire:	
Employment status, *n* (%)	
Full‐time (≥35 h/week); part‐time (<35 h/week)	68 (58.60); 47 (40.50); (1 missing)
Self‐employed	6 (5.20)
Hours worked/week, mean (SD)	34.55 (9.55)
ONS Job skill level, *n* (%)	
1 (lowest); 2; 3; 4 (highest)	7 (6.00); 47 (40.50); 30 (25.90); 32 (27.60)
Organization size (people), *n* (%)	
1 person (self)	6 (5.20)
Micro (2–9)	6 (5.20)
Small (10–49)	14 (12.10)
Medium (50–249)	15 (12.90)
Large (≥250)	75 (64.60)
Occupational health available main job (yes), *n* (%)	68 (58.60)
Disclosed condition to employer/supervisor, *n* (%)	108 (93.1)
WLQ ‐25 (0–100):	25.00 (15.00–46.73)
RA‐WIS (0–23)	16.43 (13.14–19.71)
WALS (0–36)	11.50 (8.00–16.00)
Physically demanding job (1–5)	3.00 (1.25–4.00)
Mentally demanding job (1–5)	4.50 (4.00–5.00)
Stressful job (1–5)	4.00 (3.00–5.00)

*Note*: For health and work measures, a higher score indicates worse status.

Abbreviations: DMARD, disease modifying anti‐rheumatic drug; IA, inflammatory arthritis; ISCED, International Standard Classification of Education (Low = ISCED 0–2, no formal qualifications through to lower secondary education, e.g., GCSE; Medium = ISCED 3–4, upper secondary, and post‐secondary non‐tertiary education, e.g., A Levels, BTEC, City & Guilds; High = ISCED 5–8, short cycle tertiary (e.g., diploma), Bachelor/Master/PhD degrees); NHS, National Health Service Rheumatology departments; ONS, Office for National Statistics, Skill level: 1 = elementary occupations (e.g., cleaner, refuse operative), 2 = administrative, caring, leisure, sales, customer service, process, plant, and machine operatives, 3 = skilled trades, associated technical and professional, 4 = professional, and managerial; PsA, psoriatic arthritis; RA, rheumatoid arthritis; RAID, Rheumatoid Arthritis Impact of Disease scale; RA‐WIS, RA Work Instability Scale; SF12v2, Short‐Form Health Survey 12 item version 2; WALS, Workplace Activity Limitations Scale; WLQ‐25, Work Limitations Questionnaire‐25 items.

### 3.2 Revising the UK WES‐RC

Workplace barrier frequencies are shown in Table 2. Of the 142 listed items, 18 were reported by ≤5 participants (5%). Of these 16 were removed, as these could either be considered: under an existing item (e.g., the “using staff/public toilets” barrier can include turn taps, low toilet, and access to disabled toilet); or could be recorded in the “other” option. Two were retained: “emergency evacuation route” (Section 3), for safety reasons as employees with a disability should have this; and “no modified or light work available” (Section 6). As the trial excluded patients on long‐term sick leave, it was less likely that participants would report this problem. This item is important for return‐to‐work VR, for which the WES‐RC can also be used. Most “other” barriers could be recoded into existing items. Of those remaining, none were reported by >5 participants, and could continue to be reported as “other.”

**TABLE 2:** Frequency of workplace barriers reported as sometimes or always a problem in the WES‐RC (Sections 2–7).

Workplace Barriers	*n* = 116	%
Section 2: Get ready/travel		
Getting ready for work:		
Extra time needed to dress, prepare breakfast	76	65.50
Get out of bed	47	40.50
Manage stairs	34	29.30
Get children/other family members/pets ready	20	17.24
Travel to/from/for work:		
Driving	70	60.30
Hold/turn steering wheel	25	21.60
Get in/out vehicle	20	17.20
Turn head for rear view	18	15.50
Sit for long time driving	16	13.80
Driving for work^a^	15	12.90
Shift gears	13	11.20
Stay alert/concentrate whilst driving	12	10.30
Clear ice/snow off car in winter	10	8.60
Manage car park barriers^a^	6	5.20
Turn car key in ignition^b^	5	4.30
Drop off/pick up children or others^b^	5	4.30
Other	21	18.10
Stress of travelling	24	20.70
Lifting/carrying items during travel	26	22.44
Time/energy use travelling	16	13.80
Travel for work (e.g., between sites, visiting clients)	7	6.00
Walking to work	7	6.00
Public transport	6	5.20
Section 3: Workplace access		
Get into/around workplace:		
Open doors	30	25.90
‐Managing weight of doors	17	14.70
‐Manage keypads/door locks^a^	11	9.50
‐Turn doorknobs/handles	8	6.90
Manage stairs	27	23.20
Walk round workplace	20	17.20
Parking	17	14.65
Using workplace Facilities:		
Using staff/public toilets	14	12.10
Turn taps^a^ ^,^ ^b^	4	3.40
Access to disabled toilet facilities^a^ ^,^ ^b^	4	3.40
Low toilet^b^	2	1.70
Other (e.g., access, flushing)	5	4.30
Access to staff canteen/food venue	11	9.50
Emergency evacuation route^c^	3	2.60
Section 4A: Completing job activities: Physical job demands		
Mobility:		
Standing/Prolonged standing	66	56.90
Prolonged sitting	65	56.00
Bend/kneel/squat/pick things up from low places	62	53.40
Lift, pull, push, move (equipment/people)	58	50.00
Carrying	53	45.70
Reach, raise arms above shoulders, or hold objects up	38	32.80
Get up/down from sitting	49	42.20
Climbing (e.g., ladders)	9	7.80
Hand Use:		
Writing	42	36.20
Hold items (e.g., tools/telephone)	40	34.50
Handle objects (e.g., turn pages, use mobile phone, chop food etc).	35	30.20
Pick items up	22	19.00
Hands get cold	24	20.70
Physical Actions:		
Doing repetitive activities	48	41.40
Strength/endurance whilst working	38	32.80
Body positioning issues (e.g., work in awkward spaces, workstation height/position, work whilst turning (e.g., teach, demonstrate)	35	30.20
Being able to move quickly	24	20.70
Using computers/keyboard devices:	58	50%
Typing/keyboarding/using mouse	51	44.00
Computer/laptop positioning (e.g., screen/chair/desk height/neck/back)	27	23.20
Hold/turn papers whilst typing	7	6.00
Senses:		
Vision (e.g., seeing well enough, dry eyes, blurred vision)	21	18.10
Hearing for example, hearing others)	10	8.70
Talking^b^	3	2.60
Section 4B: Completing job activities: Mental/Time/Other demands		
Time, energy, emotional job demands:		
Working extra or overtime hours	42	36.20
Meeting deadlines, production quotas or performing under stress	38	32.80
Emotional demands of working with customers/children, etc.	33	28.40
Work pace or scheduling	32	27.60
Working your regular hours	32	27.60
Starting work activities soon after getting to work	18	15.50
Working shift hours	11	9.50
Mental job Demands:		
Focusing/concentrating on work activities	54	46.60
Remembering	48	41.40
Staying alert or sustaining attention	46	39.70
Thinking quickly	23	19.80
Planning/organising	18	15.50
Other: Being a lone worker^a^ (some/all of time)	24	20.70
Section 5: Relationships with people at work (supervisors, Co‐workers, supervisees, customers, people you teach/care for)		
Supervisor‐ related issues:		
Supervisor or management not supportive	44	37.90
You fear being thought of as less valuable	23	19.80
You are treated differently, or not in the way you want	19	16.30
You are unable to explain your condition	10	8.60
Co‐worker related issues:		
Co‐workers are not supportive	35	30.20
Feel guilty about taking time off	24	20.70
You don't want to/afraid to ask for help	12	10.30
Co‐workers resent you taking time off	7	6.00
Co‐workers don't help when ask for it	6	5.20
Yours and other's reactions:		
Feeling self‐conscious about health, limitations and/or appearance	35	30.20
Lack of understanding from others about your limitations	28	24.10
Afraid/hesitant to ask for job accommodations	24	20.70
Being pleasant/upbeat with others when in pain or tired	24	20.70
Feeling the need to hide your condition from others	18	15.50
Explaining, or handling reactions of others to, your health, limitations, or appearance	12	10.30
Others don't value your role/contribution at work^a^	8	6.90
Negative reactions of people you supervise to your health	6	5.20
Wearing the right kind of clothes, uniform and/or shoes for your work	6	5.20
Section 6: Environmental factors & company policies		
Environmental factors:		
Lighting		
Fluorescent light	14	12.10
Sunlight (working outdoors)	6	5.20
Low/dim light^b^	1	0.90
Other (glare, lack of natural light)	3	2.60
Cold temperature or draughts		
Cold areas at work (e.g., cold storage)^a^	20	17.20
Cold (work outdoors)	9	7.80
Cold (air‐conditioning)	8	6.90
Other environmental Issues:		
Heat	24	20.70
Noise^a^	12	10.30
Flooring^a^ ^,^ ^b^	5	4.30
Humidity^b^	4	3.40
Smoke/other fumes/scents/dust/poor air quality^b^	3	2.60
Company policies:		
Sick Leave related issues		
No/not enough sick days	17	14.70
Supervisor frowns on use of sick days	14	12.10
No or not enough flexibility in, or exemption from, sickness absence policy if have a long‐term condition^a^	11	9.50
Limited or no company sickness benefit/pay^a^	9	7.80
Needing to take a lot of sick days^b^	5	4.30
Other (e.g., does not like to take sick days; no one else does work)	6	5.17
Other Company Policies		
Not enough chance to take some rest breaks	26	22.40
Employer is not supportive about job accommodations	18	15.50
No or not enough time off for health care appointments	15	12.90
Not enough flexibility in hours	14	12.10
No or not enough access to occupational health and/or personnel, human resources support^a^	12	10.30
No or not enough discussion of Fit Note (or return to work interview) following sick leave^a^	10	8.60
No or not enough performance reviews^a^	10	8.60
Limited or no company sickness benefit/pay^a^	9	7.80
Needing to arrive at a certain time	8	6.90
Not enough flexibility in changing shift patterns^a^	7	6.00
Not enough chance to do some work at home	6	5.20
No modified or light work available (e.g., following discussion of Fit Note)^a^ ^,^ ^c^	4	3.40
Difficulty meeting targets from performance reviews^a^ ^,^ ^b^	4	3.40
Lack of company retirement benefits^b^	0	0
Section 7: Job. Career and home life		
Job ability:		
Getting the work for your job done	61	52.60
Completing tasks as quickly as other do	34	29.30
Concern about meeting expectations	34	29.30
Loss of self‐confidence about work	26	22.40
Considering what work you would do, if you wanted or needed to change jobs	24	20.70
Having the drive needed for promotions	13	11.20
Lack of friendly relationships at work^b^	5	4.30
Job satisfaction:		
You are unhappy with your job because of your job conditions	25	21.60
Job does not give feeling of accomplishment, or opportunity for advancement	13	11.20
Low pay	9	7.80
Not enough feedback about how well you do your job	7	6.00
Job does not provide for steady employment^b^	3	2.60
You are unhappy with your job because of your health	18	15.50
You want or need to change job or career	14	12.10
Balance between work and home life:		
Self‐managing your arthritis, for example, taking medications, getting rest, exercise etc	39	33.60
Getting household work and/or shopping done	34	29.31
Doing things with your children, or other family, social, sport or recreational activities	31	26.70
Lack of family support	7	6.00
Doing volunteer activities^b^	1	0.90

^a^ Items added to the UK‐WES‐RC in 2011, during its adaptation from the United States WES‐RC.

^b^ Items now omitted from WES‐RC as <5% identified this barrier as a problem.

^c^ <5% identifying as a problem, but retained due to safety reasons (i.e., evacuation route) or because few people with this problem recruited due to trial exclusion criteria (i.e., no modified or light work available).

In the WES‐RC, some sections have items forming sub‐headings to check, followed by a list of related items (e.g., Section 2 included “Driving,” with a comprehensive sub‐list of driving problems). To avoid “double counting,” the check options for such sub‐headings were removed. This reduced items to 121, with 22 “other” options across sections for additional problems to be recorded.

For content validity, 73 of the 90 items in the ICF‐VR were linked to the WES‐RC (Supplementary Table S1). Of those 17 not included, 14 were environmental factors. Some of these were considered problematic to use in the clinical setting, as their ICF definitions are too broad. These form the health, social and economic context in which JRVR is provided, which VR providers should consider, rather than forming part of an individual assessment (Finger et al., 2012). Eight of the 12 work contextual factor domains could also be linked (Boonen et al., 2021). Three domains were then added to Section 1 Work History: workplace organisation (Q14: workload covered by others if off sick); economic need (Q17: financial concerns); and workplace accommodations, by including a reminder to record in the WES‐RC which of these people already have. The one domain not included was the economic climate/labour regulations, a societal level factor, which HCPs/OHPs should consider when providing VR, for example, social security benefits available to help support working and employment rights of working people with long‐term conditions (Supplementary Table S3). A review of additional comments recorded in the WES‐RCs by therapists also led to additional items in Section 1 Work History, about size of employing organisation, disclosure of the health condition at work, and availability of human resources department and/or occupational health in the workplace. These items provide additional context for planning JRVR.

To support therapists in formulating clear work problems to address in Section 8 (problem prioritization and solution development), several examples were included in the instruction page for Section 8, with a reminder to refer to the WES‐RC manual. The updated WES‐RC for the UK is in Supplementary File 1.

### 3.3 Psychometric properties of the UK version of the WES‐RC

Using this revised version of the UK WES‐RC (Sections 2–7: 121 barriers), internal consistency was Cronbach's α = 0.92, that is, excellent, and suitable for both individual and group use. On average, participants reported 23.00 (15.00–31.00) workplace barriers (range 4–85). Concurrent validity was moderate with the WLQ‐25 summed score (*r*_*s*_ = 0.40) and WALS (*r*_*s*_ = 0.43), but weak with the RA‐WIS (*r*_*s*_ = 0.31), although all significant (*p* < 0.001).

Mapping the work measures onto the WES‐RC highlighted that the WLQ‐25 has items related to Sections 3, 4A, 4B, and part of 7 (job ability), the WALS to Sections 4A and 4B, and the RA‐WIS to Sections 1, 4A, 4B, and 7 (job ability), with one item only for each Sections 2, 3 and 6 (Supplementary Table S3).

For discriminative validity, there were significant differences between numbers of workplace barriers reported between those with low, medium, and high risk of work disability based on their RA‐WIS and WALS scores, but not between those with differing levels of health status (Table 3). (Unexpectedly, as participants had to score ≥10 on the RA‐WIS to be eligible, 13/116 (11.20%) scored <10 when completing their baseline questionnaire).

**TABLE 3:** Discriminant validity of the UK Work Experience Survey‐ Rheumatic Conditions.

	Work instability/risk of work disability group			H	df	*p*
	Low	Medium	High			
RA‐WIS score range	0–9	10–17	18–23			
*n*	13	49	54			
No. WES‐RC barriers, median (IQR)	15.00 (10.00–26.00)	20.00 (15.50–29.00)	25.00 (16.75–37.25)	6.72	2	0.03
WALS score range	0–6	7–13	14–36			
*n*	19	55	42			
No. WES‐RC barriers, median (IQR)	14.00 (10.00–23.00)	19.50 (15.00–29.00)	29.00 (22.50–39.00)	16.85	2	<0.001

	SF‐12v2 general health group			H	df	*p*
	Excellent/Very Good/Good	Fair	Poor			
SF‐12v2 general health score	1–3	4	5			
*n* (missing = 1)	43	50	22			
No. WES‐RC barriers, median (IQR)	21.00 (13.00–30.00)	23.00 (14.50–30.00)	24.50 (17.75–38.25)	0.61	2	0.74

### 3.4 Health symptoms reported in the WES‐RC

In Section 1, over 90% reported that pain and fatigue impacted their work, with poor sleep and stress reported by around two‐thirds (Table 4). Participants reported 4.00 (IQR 3.00–5.00) of the seven symptoms listed affected their work, with women tending to report more symptoms than men, although not significantly so (*p* = 0.09). As expected, those with poor health reported more symptoms affecting work than those with fair or good health (H = 7.39; df = 2; *p* = 0.02), as did those with medium or high work instability compared with those with low instability (H = 11.98; df = 2; *p* = 0.003). There were no differences in the number of health symptoms reported by people with RA, early IA, or PsA (H = 1.45; df = 2; *p* = 0.48).

**TABLE 4:** Health symptoms reported in Section 1 of the WES‐RC as a problem in regard to work.

	*n* = 116	%
Pain	110	94.80
Fatigue or low energy	107	92.24
Poor sleep/irritability	78	67.20
Stress/nervousness/worry	75	64.70
Depression/anxiety	52	44.80
Sudden changes in symptoms and ability to do things	49	42.20
Medication side effects causing a problem at work	16	13.80

### 3.5 Workplace barriers reported in the WES‐RC

About a quarter of barriers (*n* = 35) were reported as “sometimes or always a problem” by ≥25% of participants, and half (*n* = 53) by ≥20% (Table 2). The number of workplace barriers reported was moderately correlated with the number of health symptoms (*r*_*s*_ = 0.50; *p* = 0.001). There were no differences in the number of workplace barriers reported by people with RA, early IA, or PsA (H = 2.23; df = 2; *p* = 0.33). Women reported more barriers (23.50 (IQR 16.25–33.75)) than men (18.50 (IQR 11.25–27.50); *p* = 0.05).

The frequency with which WES‐RC sections and sub‐sections were reported as problematic, and the average number of barriers and frequency of major barriers within each, is reported in Table 5. On average, workplace barriers in 5.00 (IQR 4.00–6.00) out of the seven sections were reported (with Section 4A and 4B considered as two separate sections in analysis). Of these, participants reported major problems in 3.00 (IQR 2.00–4.00) sections.

**TABLE 5:** Frequency of barriers identified in each UK WES‐RC section (*n* = 116).

	WES section	No. items in section	*n* (%) reporting problem(s) in section always/sometimes	Median (IQR) no. items reported by those with problems	Major problem/s in section identified. *n* (% of those with problem(s))	% with major problem/s (*n* = 116)
2	Get ready/Travel to, from and for work	**19**	**101 (87.10)**	**4.00 (1.00 – 6.00)**	**36 (35.64)**	**31.03**
‐Get ready		4	86 (74.10)	1.50 (0–2.00)	17 (19.76)	14.66
‐Travel		15	84 (72.40)	2.00 (0–3.75)	24 (28.57)	20.68
3	Workplace access	**9**	**60 (51.70)**	**1.00 (0 – 2.00)**	**16 (26.66)**	**13.70**
‐Get around		6	58 (50.00)	0.5 (0–1.00)	17 (29.31)	14.65
‐Workplace facilities		3	20 (17.20)	0 (0–0)	5 (25.00)	4.31
4A	Completing job activities physical	**23**	**116 (100)**	**7.00 (5.00 – 10.00)**	**89 (76.72)**	**76.72**
‐Mobility		8	115 (99.10)	3.00 (2.00–5.00)	61 (53.01)	52.51
‐Hand use		5	86 (74.10)	1.00 (0–2.00)	35 (40.69)	30.17
‐Physical actions		4	77 (66.40)	1.00 (0–2.00)	31 (40.26)	26.72
‐Computer use		4	58 (50.00)	0.50 (0–2.00)	34 (58.62)	29.31
‐Senses		2	30 (25.90)	0 (0–0.75)	4 (13.33)	3.44
4B	Completing job activities: Mental, time, energy, emotional, lone worker	**13**	**106 (91.40)**	**3.00 (2.00 – 5.00)**	**63 (59.43)**	**54.31**
‐Time, energy, emotional demands		7	90 (77.60)	2.00 (1.00–3.00)	48 (53.33)	41.37
‐Mental job demands		5	78 (67.24)	1.00 (0–2.75)	32 (41.03)	27.50
‐Lone worker		1	24 (20.70)	0 (0–0)	1 (4.16)	0.09
5	Relationships at work	**18**	**74 (63.80)**	**2.00 (0 – 5.00)**	**45 (60.81)**	**38.79**
‐Supervisor		4	44 (37.90)	0 (0–2.00)	27 (61.36)	23.17
‐Co‐workers		5	35 (30.20)	0 (0–2.00)	14 (40.00)	12.07
‐Others' perceptions, self‐perceptions.		9	60 (51.70)	1 (0–2.00)	27 (45.00)	23.27
6	Environmental factors & company policies	**23**	**86 (74.10)**	**2.00 (0 – 3.00)**	**40 (46.51)**	**34.48**
‐Environmental factors		8	59 (50.86)	1.00 (0–2.00)	9 (15.00)	7.78
‐Sick leave‐related issues		4	36 (31.00)	0 (0–1.00)	12 (33.33)	10.34
‐ Other company policies		11	57 (49.10)	0 (0–2.00)	23 (40.35)	19.82
7	Job, career & home life	**16**	**89 (76.70)**	**3.00 (1.00 – 5.00)**	**59 (67.82)**	**50.86**
‐Job ability		4	70 (60.30)	2.00 (0–3.00)	19 (31.15)	16.38
‐Job satisfaction		6	35 (30.20)	0 (0–1.00)	12 (34.28)	10.34
‐Balance work and home life		4	63 (54.30)	1.00 (0–2.00)	42 (66.66)	36.21
TOTAL WES‐RC		**121**	**116 (100)**	**23.00 (15.00 – 31.00)**	‐	‐

Over three‐quarters of participants reported “sometimes or always” experiencing workplace barriers in:

(i) *Section* *4* *: Completing Job Activities Part A: Physical Demands* by 100%, particularly in Mobility (99.10%), with the most frequently identified individual mobility barriers being prolonged standing, prolonged sitting, bending/kneeling/squatting, and lifting/pulling/pushing objects (50%–57%); Hand Use (74.10%); Physical Actions (e.g., repetitive actions, strength) (66.40%); and Computer Use (50%). For those with such barriers, 40%–58% identified them as major problems at work.
(ii) *Section* *4* *: Completing Job Activities Part B: Time, Energy, Emotional, and Mental Demands* by 91.40%, the most frequently identified barriers being concentrating on work, remembering, and staying alert (40%–46%), and working extra hours and meeting job deadlines (33%–36%). For those with such barriers, 41%–53% identified them as major problems at work.
(iii) *Section* *2* *: Getting Ready for and Travel to/from/for Work* by 87.10%, with extra time to dress/prepare breakfast (66%) and driving (60%) most often reported. For those with such barriers, 19%–28% identified them as major.
(iv) *Section 7: Job, Career and Home Life* by 76.70%, with the most common barriers identified being Job Ability (60%, e.g., getting the work for your job done (53%)), and Work‐life Balance barriers (54%, e.g., self‐managing arthritis (34%) and getting housework/shopping done (29%)). For those with such barriers, 31% and 66% respectively, identified them as major.

Between half and three‐quarters reported barriers in the other three sections:

(v) *Section 6: Environmental Factor and Company Policies* by 74.10%, particularly with sick leave‐related issues (31%) and other company policies (49%) (e.g., not enough chance to take rest breaks (22%)). For those with such barriers, 33% and 40%, respectively, identified them as major.
(vi) *Section 5: Relationships at Work* by 63.80%, particularly others’‐ and self‐ reactions (52%) and lack of support from supervisors (38%) and co‐workers (30%). For those with such barriers, 40%–61% identified them as major, with poor supervisor support the highest.
(vii) *Section* *3* *: Workplace Access* by 51.70%, particularly opening doors (26%) and managing stairs (23%). For those reporting access issues, it was a major barrier for 29%.

### 3.6 Updating the WES‐RC manual and Workwell Solutions Manual

The WES‐RC Manual was updated by including the revised WES‐RC. Further information was added to explain how to conduct the WES‐RC and about formulating problems in Section 8, as well as a completed sample WES‐RC. This contains sufficient information to allow health and occupational health professionals to use the UK WES‐RC in practice, outlines the range of work rehabilitation solutions possible and lists work rehabilitation resources. The Workwell Solutions Manual was revised by checking all weblinks and reviewing completed WES‐RCs, which identified new resources therapists identified during the Workwell trial. Case studies with JRVR case notes were included, based on real cases in the Workwell trial, to support health professionals in identifying solutions and implementing work rehabilitation for problems identified in the WES‐RC.

## 4 DISCUSSION

This study produced an updated UK version of the WES‐RC listing most workplace barriers reported by working people with IA. The study demonstrated for the first time that the WES‐RC has good content validity with the ICF‐VR, addresses key personal and work environmental contextual factors, has sufficient reliability (internal consistency) for individual use, as well as group use, and good discriminative validity in IA. It was a priori hypothesised that concurrent validity of the number of WES‐RC barriers with work and health measures would be moderate at best, which was the case. Mapping the content of the work measures indicated why. All three of the work measures have little, or no, coverage of items related to barriers in Sections 2 (getting ready for and travel to work), 5 (relationships with people at work: supervisors, co‐workers, others), 6 (environmental factors and company policies) and part of 7 (specifically, job satisfaction and work‐life balance). Yet, between 54% and 87% of participants reported barriers in these sections. This highlights the importance of going beyond using short work outcome measures, and the importance of using a comprehensive biopsychosocial assessment to assess work barriers and plan VR.

Over a third of the work barriers listed in the WES‐RC were reported as problematic by 25% or more of participants, with the most common being physical problems of prolonged standing, sitting, bending/kneeling/crouching, lifting, and using hands whilst working. Studies investigating work limitations in IA using WALS, similarly identified these as the most common (Brown et al., 2023; Gignac et al., 2011; Xavier et al., 2019).

Workplace barriers experienced by RMD participants (*n* = 143) in the WORK‐IT trial (Keysor et al., 2018) have also been reported (AlHeresh et al., 2023). Participants were predominantly diagnosed with osteoarthritis (43%), RA (23%), chronic back pain (13%), fibromyalgia (11%), and other RMDs. The most common barriers were also in Section 4A Physical Job Demands, of prolonged sitting, prolonged standing, and bending/kneeling (52%–55%). Hand function‐related barriers were reported by less than a third, compared with three‐quarters in this study. This reflects the higher percentage of participants with RA, as typically over 90% report hand and wrist involvement, even with low disease activity (Horsten et al., 2010). In WORK‐IT, the second most common barriers were in Section 2 Getting Ready for, and Travel to/from Work (38%–44%). This was the third most common in this study, with a higher percentage reporting such barriers, highlighting the importance of considering barriers outside the workplace in VR. In this study, Section 4B, completing mental, time, energy and emotional demands, was the second most common section with problems, compared to less than 25% in WORK‐IT, reflecting that 31%–72% of people with RA experience cognitive impairment (McDowell et al., 2022; Shin et al., 2012).

In the WORK‐IT study, the third most common section with problems was Section 5 Relationships with People at Work. The most common problems were feeling self‐conscious about health at work, hiding their condition from others, needing to be pleasant when in pain, and afraid to ask for work accommodations (each 37%). In this study, two thirds also experienced barriers in Section 5, but these differed in nature and frequency. Unsupportive relationships with supervisors or co‐workers were each reported by around a third. Poorer supervisor support is associated with worse employee physical and mental well‐being, job satisfaction, job stress and increased job demands (Hämmig, 2017; Chartered institute of Personnel Development (2023). These findings emphasise the importance in JRVR of facilitating communication between employee and manager about problems at work, particularly in understanding the impact of RMDs on work, and in requesting work accommodations.

Presenteeism is influenced by condition‐related factors of reduced physical function, greater fatigue, pain, and disease activity, and personal and work‐related factors of poorer mental health, negative impact of IA on work and work‐life balance, difficulty managing the mental and interpersonal demands of their job, higher levels of job stress, and the number of work accommodations needed (Boot et al., 2018; Brown et al., 2023; Druce et al., 2018; Gwinnutt et al., 2020; Kim et al., 2017; Xavier et al., 2019). This study highlighted that the WES‐RC assesses these factors and the frequency with which such problems are being experienced by those needing JRVR.

### 4.1 Strengths and limitations of the study, and future research

A strength of the study was that participants were those most likely to need JRVR, as a medium to high level of work instability was required to be eligible. Participants represented a wide range of educational backgrounds, job skill levels (although few at level 1: essential jobs) and organisation size. A limitation is that, as they volunteered to participate in a trial, particularly as part of this occurred during the COVID‐19 pandemic (which understandably led to fewer patients wanting to participate), they may not fully represent all those with IA benefitting from JRVR.

As the WES‐RC is a clinical assessment, some psychometric properties, such as identifying structural validity using Rasch analysis, were not applicable to investigate. Further research is needed to investigate test‐retest and inter‐rater reliability. The WES‐RC could potentially be used as a self‐report assessment, although criterion validity should be established, with the criterion being the WES‐RC conducted by VR‐experienced therapists to ensure that barriers are fully reported. Working people with health conditions may not always want to admit to themselves they have workplace difficulties, as it may undermine their sense of identity (British Society of Rehabilitation Medicine, 2022), meaning self‐report may not uncover the full extent of problems. If used as self‐report, whilst establishing a therapeutic relationship, therapists can briefly discuss each WES‐RC section to ensure all barriers are identified prior to problem and treatment plan formulation. If used as self‐report, people with RMDs could also use the Workwell Solutions Manual themselves to identify work solutions. An online version of the WES‐RC and Workwell Solutions Manual will be available in the future to facilitate this.

### 4.2 Conclusion

The study demonstrated that WES‐RC has acceptable validity and good reliability for individual clinical use, as well as for group use. The study highlighted the wide range of workplace barriers experienced by working people with IA, emphasising the need to provide individualised biopsychosocial JRVR.

The WES‐RC, WES‐RC Manual, and Workwell Solutions Manual are freely available for download and use in clinical practice and research (Hammond et al., 2023b, 2023c, 2023d).

## AUTHOR CONTRIBUTION

**Alison Hammond**: Conceptualization, funding acquisition, methodology, formal analysis, writing – original draft, writing – review and editing, resources. **Rachel O’Brien**: Funding acquisition, writing – review and editing, resources. **Sarah Woodbridge**: Funding acquisition, writing – review and editing, resources. **Jennifer Parker**: Project administration, data curation, formal analysis, writing – review and editing. **Angela Ching**: Project administration, data curation, writing – review and editing.

## CONFLICT OF INTEREST STATEMENT

The authors have no conflicts of interest to report.

## ETHICS STATEMENT

This study was performed in line with the principles of the Declaration of Helsinki. Approval was granted by the National Research Ethics Service Committee West Midlands – Solihull Research Ethics Committee (18/WM/0327), and the University of Salford's School of Health & Society Ethics Panel (HSR 1819‐010). All participants provided informed, written consent.

## TRIAL REGISTRATION

Data were collected as part of the WORKWELL Trial: ISRCTN: 61762297; Clinical Trials.Gov: NCT03942783.

## Supporting information

Supporting Information S1

## Data Availability

The data that support the findings of this study are available from the corresponding author upon reasonable request.
