# Supplementary material for: The Work Experience Survey – Rheumatic conditions (United Kingdom): Psychometric properties and identifying the workplace barriers of employed people with inflammatory arthritis receiving vocational rehabilitation
Source: Musculoskeletal Care. 2023 Oct 25;21(4):1578–91. doi: 10.1002/msc.1835 (PMC10946830; doi:10.1002/msc.1835)
Supplement: Supplementary file 1 — Supporting Information S1 [file MSC-21-1578-s001.pdf]

Supplementary Materials:

The Work Experience Survey – Rheumatic Conditions (United Kingdom): psychometric properties and identifying workplace barriers of employed people with inflammatory arthritis receiving vocational rehabilitation.

Hammond A, O'Brien R, Woodbridge S, Parker J, Ching A. Musculoskeletal Care 2023

<u>Table of Contents</u>

		**Pages**
Supplementary Table 1	The Comprehensive ICF Core Set for Vocational Rehabilitation linked to the Work Experience Survey – Rheumatic Conditions.	**2-4**
Supplementary Table 2	Contextual factors influencing worker productivity (Boonen et al, 2021).	**5**
Supplementary File 1	The Work Experience Survey – Rheumatic Conditions (United Kingdom version).	**6-22**
Supplementary Table 3	Contextual factors domains and Items from the WLQ-25, WALS and RA-WIS linked to WES-RC Sections 1 – 7.	**23-27**

Supplementary Table 1: The Comprehensive ICF Core Set for Vocational Rehabilitation linked to the Work Experience Survey – Rheumatic Conditions.

**ICF Code**	**ICF Category Title**	**WES-RC Section linked to code/category**
**Activities and Participation (40)**		
d155	Acquiring skills	[4B Mental job demands]
d160	Focusing attention	4B Mental job demands
d163	Thinking	4B Mental job demands
d166	Writing	4A Hand Use
d172	Calculating	[4B Mental job demands]
d175	Solving problems	[4B Mental job demands]
d177	Making decisions	[4B Mental job demands]
d210	Undertaking a single task	[4B Time demands]
d220	Undertaking multiple tasks	[4B Time demands]
d230	Carrying out daily routine	2
d240	Handling stress and other psychological demands	4B Time Energy Emotional Demands
d310	Communicating with - receiving - spoken messages	4A Senses
d315	Communicating with - receiving - nonverbal messages	4A Senses
d350	Conversation	4A Senses
d360	Using communication devices and techniques	4A Computers/ Hand Use
d410	Changing basic body position	4A Mobility/ Physical Actions
d415	Maintaining a body position	4A Mobility// Physical Actions
d430	Lifting and carrying objects	4A Mobility
d440	Fine hand use	4A Computers/ Hand Use
d445	Hand and arm use	4A Mobility
d450	Walking	3
d455	Moving around	3
d465	Moving around using equipment	4A Mobility
d470	Using transportation	2
d475	Driving	2
d530	Toileting	3
d540	Dressing	2
d570	Looking after one’s health	7 Balance between work and home life
d710	Basic interpersonal interactions	5
d720	Complex interpersonal interactions	5
d740	Formal relationships	5 Supervisor
d820	School education	1 Q5

d825	Vocational training	1 Q6
d830	Higher education	1 Q5
d840	Apprenticeship (work preparation)	1 Q6
d845	Acquiring, keeping, and terminating a job	1 Work History
d850	Remunerative employment	1 Work History
d855	Non-remunerative employment	
d870	Economic self-sufficiency	1 Work History
**Environmental Factors (33)**		
e11001	Drugs	1 Health
e115	Products and technology for personal use in daily living	2
e120	Products and technology for personal indoor and outdoor mobility and transportation	2 and 3
e125	Products and technology for communication	4A Computer Use; Hand Use
e130	Products and technology for education	
e135	Products and technology for employment	4A
e150	Design, construction and building products and technology of buildings for public use	3 Workplace Access
e155	Design, construction and building products and technology of buildings for private use	[2 Home access]
e225	Climate	6 Environmental Factors
e240	Light	6 Environmental Factors
e250	Sound	6 Environmental Factors
e260	Air quality	6 Environmental Factors
e310	Immediate family	7 Balance Work and Home Life
e320	Friends	7 Balance Work and Home Life
e325	Acquaintances, peers, colleagues, neighbours, and community members	5 Co-workers; 7 Balance Work and Home Life
e330	People in positions of authority	5 Supervisors/ Management
e340	Personal care providers and personal assistants	
e355	Health professionals	1 Health
e360	Other professionals	1 Work History Q15
e430	Individual attitudes of people in positions of authority	5 Supervisors/ Management
e450	Individual attitudes of health professionals	
e460	Societal attitudes	
e465	Social norms, practices, and ideologies	
e525	Housing services, systems, and policies	

e535	Communication services, systems, and policies	
e540	Transportation services, systems, and policies	
e550	Legal services, systems, and policies	
e555	Associations and organizational services, systems, and policies	
e565	Economic services, systems, and policies	
e570	Social security services, systems, and policies	
e580	Health services, systems, and policies	
e585	Education and training services, systems, and policies	
e590	Labour and employment services, systems, and policies	6 Company Policies (local level)
**Body Functions (17)**		
b117	Intellectual functions	4B Mental job demands
b126	Temperament and personality functions	5 Your reactions
b130	Energy and drive functions	1 Health
b134	Sleep functions	1 Health
b140	Attention functions	4B Mental job demands
b144	Memory functions	4B Mental demands
b152	Emotional functions	4B Emotional job demands
b160	Thought functions	4B Mental job demands
b164	Higher-level cognitive functions	4B Mental job demands
b210	Seeing functions	4A Senses
b230	Hearing functions	4A Senses
b235	Vestibular functions	
b280	Sensation of pain	1 Health
b455	Exercise tolerance functions	4A Physical activity
b730	Muscle power functions	4A Physical activity
b740	Muscle endurance functions	4A Physical activity
b810	Protective functions of the skin	

Supplementary Table 2: Contextual factors influencing worker productivity (Boonen et al, 2021)..

Personal Contextual Factors	Environmental Contextual Factors
**1. Health:**<br>• Pain<br>• Fatigue<br>• Physical Function	**7. Nature of work:**<br>• Physical/ mental demands<br>• Job autonomy
**2. Demographics:**<br>• Age and gender<br>• Education	**8. Workplace Support:**<br>• Assistance by co-workers<br>• Attitude of employer
**3. Economic Need:**<br>• Income needs<br>• Quality of benefits	**9. Workplace organisation:**<br>• Team dynamics at work<br>• Compensation of work absences (e.g., by replacement workers)
**4. Personal Appraisal of work:**<br>• Job satisfaction<br>• Career perspectives	**10. Workplace accommodations**<br>• Adaptive devices<br>• Modified hours/ duties
**5. Related skills/ abilities:**<br>• Work self-efficacy<br>• Coping skills	**11. Economic climate/ labour regulation**:<br>• Income compensation<br>• Employment opportunities
**6. Work-life balance:**<br>• Competing social roles<br>• Quality of leisure	**12. Non-workplace support:**<br>• Support from family<br>• Task assistance at home

WORKWELL

KEEPING PEOPLE WORKING

THE WORK EXPERIENCE SURVEY

for

People with Rheumatic Conditions

(WES-RC): UK Version.

© Alison Hammond, Sarah Woodbridge, Rachel O'Brien 2023. **DOI: 10.13140/RG.2.2.27973.01764 CC-BY**

The UK version was adapted by the authors from the Work Experience Survey for Persons with Rheumatic Conditions with kind permission from Prof Saralynn Allaire, Boston University, Boston, MA, USA.

The UK WES-RC may be used in clinical practice and research. If reproduced in any form (e.g., paper, electronic), the authors and Prof Allaire must be acknowledged.

Please read: instructions in the UK WES-RC Manual (2023) before using assessment
DOI 10.13140/RG.2.2.24424.24329

For solutions to identified problems -see the Workwell Solutions Manual (2023)

Go to [https://www.researchgate.net/profile/Alison-Hammond-2/research/ Technical Reports](https://www.researchgate.net/profile/Alison-Hammond-2/research/) to download.

The revision of the WES-RC (UK) was funded by Versus Arthritis.

Preliminary Information

Section 1. Demographic, Health and Work History Information

Demographics

1. Age ______ 2. Gender_____ 3. Marital/family status ______________________

4. Highest level of formal education received:

☐ No formal educational qualifications

☐ CSE/ O Level/ GCSE

☐ City & Guilds/ GNVQ: Level: ______________

☐ A Level/ BTEC

☐ Diploma

☐ Degree

Other_________________________

5. Other vocational/ professional training (details): ___________________________________

Health

6. Primary rheumatic condition (diagnosis) ____

7. Number of years has had primary rheumatic condition _________

8. Other health conditions/disabilities ____

9. Medications ____

____

10. **Health symptoms or issues: check any that are a problem in regard to work**.

☐ Pain

☐ Fatigue or low energy

☐ Sudden changes in symptoms and ability to do things.

☐ Stress/ nervousness/ worry

☐ Poor sleep/ irritability

☐ Depression/ anxiety

☐ Medication side effects causing a problem at work (describe) ________________________

☐ Other (describe) ____

11. Regular rheumatology/ medical care? ____

Complete the diagram below to identify which joints/areas are problematic.

Note any specific problems identified e.g., R knee OA; L de Quervain's.

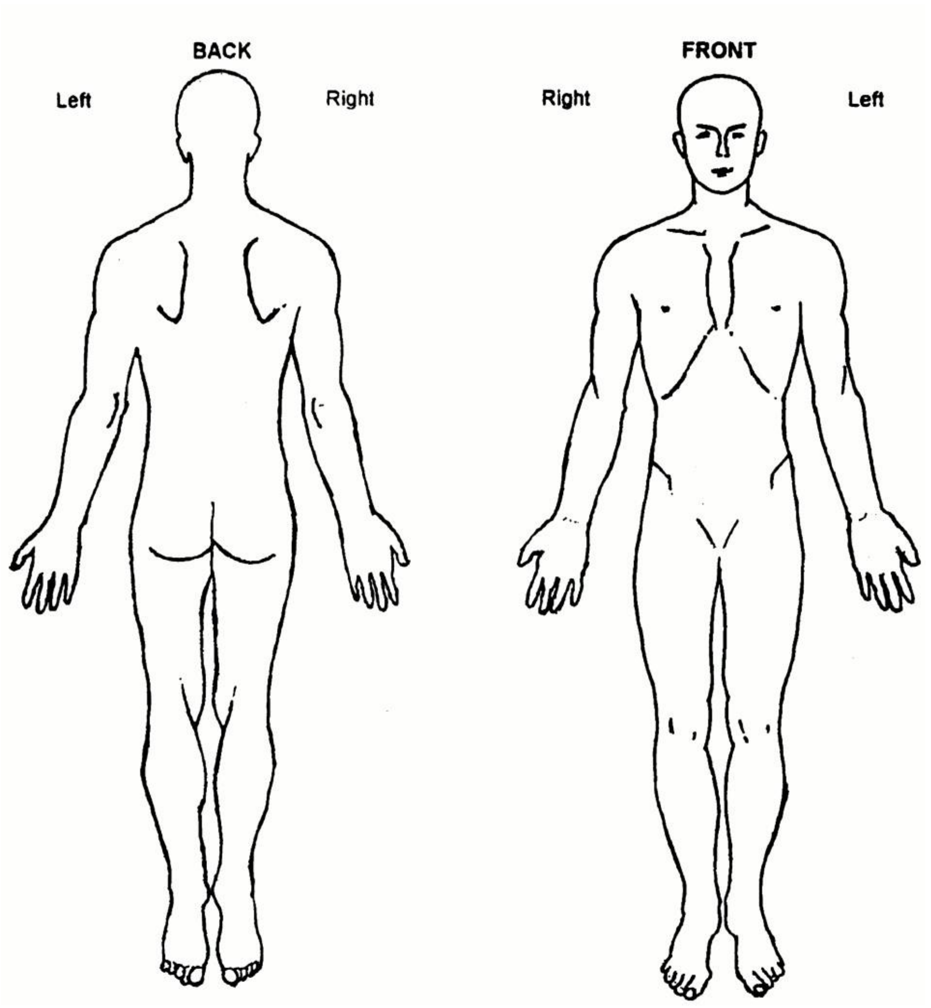

Additional Notes

Work History

1. Number of jobs held currently _______

2. Self-employed? Yes☐ No☐

3. Title of main job

____

4. List 3 activities performed regularly in main job

a. ____

b. ____

c. ____

5. Number of years worked in main job _______________

6. If less than 5 years, previous jobs held: ____

____

7. Number of hours worked per week in all jobs_________

8. Normal working hours/ days ____

9. Do you do shift work? Yes☐ No ☐

10. If yes, pattern of shift hours: ____

11. What size of company do you work for?

Very small (1-9) ☐ Small (10-49) ☐ Medium (50-249) ☐ Lage (250 or more) ☐

12. Does your employer/ manager know about your health condition? Yes☐ No ☐

13. Do your co-workers know about your health condition? All ☐ Some ☐ None ☐

14. If you are off sick, does anyone else cover your work? Yes☐ No ☐

15. Human resources department/ occupational health where you work? Yes☐ No ☐

16. Any retirement issues? (e.g., plans for/ concerns about continuing to work) Yes☐ No ☐

____

17. Any financial concerns (include considering benefits)? * Yes☐ No ☐

Sections 2 – 7:

As applicable, briefly note if the client has any workplace accommodations already (e.g., adaptive devices, modified hours/ duties).

Section 2. Getting Ready for Work and Traveling to and from, or for Work

Please tick the items that are sometimes, or always, a problem for you.

Getting ready for work

☐ Getting out of bed

☐ Extra time needed for dressing, preparing breakfast, etc.

☐ Getting children, other family members or pets ready

☐ Doing stairs at home

☐ Other (describe) ____

Traveling to and from, or for work

☐ Using public transport (describe) ____

☐ Walking or cycling to work.

Driving:

- ☐ Get in and out of vehicle.
- ☐ Turn head as needed for rear view.
- ☐ Change gear.
- ☐ Hold or turn steering wheel.
- ☐ Sit a long time in car.
- ☐ Stay alert or concentrate on driving.
- ☐ Clear snow and ice in winter (from car or drive)
- ☐ Managing car park barriers
- ☐ Driving for work (describe hours) _______________________________________
- ☐ Other (describe, e.g., operate hand/foot brakes, accelerator) __________________

☐ Time/energy use

☐ Stress of getting to work on time.

☐ Travel for business (describe) ____

☐ Lifting and/ or carrying things

☐ Other (describe) ____

Are any of the items you ticked major problems? Yes ☐ No ☐

If yes, please highlight or circle items that are major. (*Major means often or fairly bothersome*)

<u>Section 3. Workplace Access</u>

Please tick the items that are <u>sometimes, or always</u>, a problem for you.

Getting into or around your place/s of work

☐ Parking (e.g., location; width of space)

☐ Walking

☐ Stairs

Opening doors:

☐ Door weight

☐ Turn doorknobs.

☐ Keypads/ door locks

☐ Other (describe) ____

Using workplace facilities

☐ Using staff/public toilets: describe (e.g., turn taps, low toilet, access to disabled toilet):
____

☐ Access to food/ eating places (e.g., staff canteen, sandwich bar)

☐ Emergency evacuation routes

☐ Other (describe) ____

Are any of the items you ticked major problems? Yes ☐ No ☐

<u>If yes,</u> please highlight or circle items that are major. (*Major means often or fairly bothersome*)

Section 4. Completing Job Activities

Please tick the items that are sometimes, or always, a problem for you.

Physical job demands:

Mobility

☐ Standing or being on feet too long

☐ Prolonged sitting (describe any chair problems) ____________________________________

☐ Getting up and down from sitting (describe) _______________________________________

☐ Lifting, pulling, pushing, or moving materials, equipment, or people

☐ Carrying things

☐ Bending, kneeling, squatting, or picking things up from low places

☐ Reaching, raising arms above shoulders, or holding objects up

☐ Climbing, for example ladders

Physical Actions

☐ Body position issues (describe, e.g., working in awkward positions)____________________

☐ Being able to move quickly.

☐ Doing repetitive activities

☐ Strength or endurance issues (describe)____

Computer/ laptop/ tablet/ other hand-held device use.

☐ Computer/laptop positioning (describe, e.g., screen/chair/desk height/neck/back)
____

☐ Typing, keyboarding, and/or using the mouse ______________________________

☐ Holding / turning / reading papers while typing

☐ Using other devices (e.g., tablets, hand-held devices, smartphone)______________

☐ Other computing issues (describe)_______________________________________

Other hand or wrist use.

☐ Holding things (e.g., tools, phone) or opening things (e.g., jars, drawers)

☐ Handling objects, e.g., turn pages, chop food, etc.

☐ Picking things up

☐ Writing

☐ Hands get cold.

☐ Other (describe)____

Senses

☐ Seeing well or other vision issues (e.g., reading, describe) __________________________

☐ Hearing or listening issues (describe) ____

☐ Other (e.g., talking, gesturing; describe) ____

Are any of the items you ticked major problems? Yes ☐ No ☐

<u>If yes,</u> please highlight or circle items that are major. (*Major means often or fairly bothersome*)

<u>Section 4. Completing Job Activities (continued).</u>

Please tick the items that are <u>sometimes, or always</u>, a problem for you.

Mental job demands.

☐ Staying alert or sustaining attention

☐ Remembering

☐ Thinking quickly

☐ Focusing or concentrating on work activities

☐ Planning or organizing

☐ Other (describe) ____

Time, Energy and Emotional job demands.

☐ Working your regular hours

☐ Working extra or overtime hours

☐ Working shift hours

☐ Starting on work activities soon after you get to work.

☐ Work pace or scheduling issues.

☐ Meeting time or production quotas or deadlines or perform under stress.

☐ Emotional demands of working with children, customers, clients, patients, etc.

☐ Other (describe)____

Any other job demands.

☐ Lone worker (some or all of time) (describe)__________________________________

☐ Other (describe)____

☐ Other (describe)____

Are any of the items you ticked major problems? Yes ☐ No ☐

<u>If yes,</u> please highlight or circle items that are major. (*Major means often or fairly bothersome*)

Section 5. Relationships with People at Work – Supervisors, Co-workers, People You Supervise, Customers, or Persons You Teach or Care For

Please tick the items that are sometimes, or always, a problem for you.

Supervisors/management

☐ Supervisor, or management, is not supportive.

☐ You are unable to explain your condition to supervisor; supervisor does not understand.

☐ You are treated differently, or not in the way you want, by supervisor /management.

☐ You fear being thought of as less valuable by supervisor or management.

☐ Other (describe)____

Co-workers

☐ Co-workers are not supportive.

☐ They don’t help when you ask for it.

☐ You don’t want/ or are afraid to ask for help.

☐ You feel guilty about taking time off, or about doing less work, due to your health.

☐ Co-workers resent you taking time off due to your health.

☐ Other (describe)____

Yours and other’s reactions

☐ Reactions of people you supervise to your health (describe) ________________________

☐ Others don’t value your role/ contribution at work.

☐ Feeling the need to hide your health condition from others.

☐ Feeling self-conscious about your health condition, limitations, or appearance

☐ Explaining or handling reactions of others to your health, limitations, or appearance

☐ Lack of understanding from others about your limitations

☐ Being afraid or hesitant to ask for a job accommodation.

☐ Being pleasant and upbeat with others when in pain or tired

☐ Wearing the right kind of clothes/ uniform or shoes for your work

☐ Other (describe) ____

Are any of the items you ticked major problems? Yes ☐ No ☐

If yes, please highlight or circle items that are major. (***Major means often or fairly bothersome***)

<u>Section 6. Environmental Factors and Company Policies</u>

Please tick the items that are <u>sometimes, or always</u>, a problem for you.

Environmental factors:

Lighting

☐ Fluorescent lights

☐ Sunlight – working outdoors.

☐ Other (describe, e.g., low/ dim light) ____

Cold temperature or draughts

☐ Air conditioning/ ventilation

☐ Cold areas at work (e.g., cold storage, draughts, cold buildings)

☐ Work outdoors.

☐ Other (describe) ____

☐ Hot or humid temperatures/ areas

☐ Noise

☐ Working environment other (describe e.g., air quality, flooring)

____

Company Policies:

Sick Leave-related issues.

☐ No or not enough sick days / taking annual leave when sick.

☐ Supervisor or management frowns on use of sick days

☐ No, or not enough flexibility in, or exemption, from company sickness absence policy if you have a long-term condition.

☐ Limited or no company sickness benefit/ pay.

☐ Other (describe) ____

Other company policies

☐ Needing to arrive at a certain time.

☐ Not enough flexibility in hours

☐ Not enough flexibility in changing shift patterns

☐ Not enough chance to take rest breaks.

☐ No or not enough time off for health care appointments.

☐ Not enough chance to do some work at home.

☐ No or not enough performance reviews.

☐ Employer is not supportive about job accommodations.

☐ No or not enough access to occupational health and/ or human resources/ personnel support

☐ No or not enough discussion of Fit Note (or return to work interview) following sick leave.

☐ No modified or light work available (e.g., following discussion of Fit Note)

☐ Other (describe, e.g., problems meeting targets/ deadlines; lack of workplace pension…)

Are any of the items you ticked major problems? Yes ☐ No ☐

<u>If yes,</u> please highlight or circle items that are major. (***Major means often or fairly bothersome***)

<u>Section 7. Job, Career and Home Life</u>

Please ticked the items that are <u>sometimes, or always</u>, a problem for you.

Job ability

☐ Getting the work for your job done.

☐ Completing tasks as quickly as others do.

☐ Concern about meeting expectations.

☐ Loss of self-confidence about your work.

☐ Having the drive or energy needed for promotions.

☐ Considering what work you would do if you needed or wanted to change jobs.

☐ Other (describe) ____

Job satisfaction

☐ You are unhappy with your job because of your health.

☐ You are unhappy with your job because of job conditions.

☐ Job does not give a feeling of accomplishment, or opportunity for advancement.

☐ Low pay.

☐ You don't get enough feedback about how well you do your job.

☐ You want or need to change jobs or career.

☐ Other (describe, e.g., lack of steady employment) _______________________________

Balance Between Work and Home Life

☐ Getting household work and/ or shopping done

☐ Lack of family / significant other support (describe) ______________________________

☐ Doing things with your (grand)children, or doing other family, social, sport and recreational activities

☐ Self-managing your arthritis (e.g., taking medications, getting rest, exercise, pacing, using ergonomics (joint protection) at work, relaxing, managing fatigue, managing stress).

☐ Other (describe) ____

Are any of the items you ticked major problems? Yes ☐ No ☐

<u>If yes,</u> please highlight or circle items that are major. **(*Major means often or fairly bothersome*)**

Section 8. Problem Prioritization and Solution Development

Review the problems identified in sections 2 through 7 with the client, which they identified as often or fairly bothersome. Remember to also consider health symptoms affecting work in section 1 (Q11) and identify work-related barriers contributing to these.

Use these to identify the **THREE** most bothersome problems/ barriers. You can group related problems together, as relevant, which may span several sections. Some examples are:

1. **Using computer/ VDU**: typing/ mouse / keyboard/ neck and arm-wrist positioning/ desk height; and office chair support/height.

2: **Getting to/from work**: driving/ operating car park barriers/ location car park space/ carrying items to office.

3. **Fatigue at work:** not enough rest breaks, problems pacing at work, awkward work positions, repetitive actions, prolonged standing, self-managing arthritis.

4. **Hand/ arm use**: lift and carry boxes, reach to high shelves, hold tools, handle work equipment), strength / endurance issues.

5. **Job stress**: difficult relationship with supervisor, getting work for job done, loss of self-confidence about work, problems planning/ organizing/ focusing on work, self-managing arthritis.

- Discuss possible solutions for the 3 problems with the client, and list these. Then note which solution/s you and the client agree are best to use, and the resources or people who can help. Be specific.
- Use this information to help both **your client and you to each write Action Plans** about what you will both do to put solutions into place.
- Refer to the WES-RC Manual to assist completing this assessment.

Problem/barrier 1:

Possible solutions considered.

Which solution/s have you / client chosen:

Resources/ people to help:

Problem/barrier 2:

Possible solutions considered:

Which solution/s have you / client chosen:

Resources/ people to help:

Problem/barrier 3:

Possible solutions considered:

Which solution/s have you / client chosen:

Resources/ people to help:

Supplementary Table 3: Contextual factors domains and Items from the WLQ-25, WALS and RA-WIS linked to WES-RC Sections 1 – 7.

	WES-RC Section	Contextual Factors, WLQ-25, WALS and RA-WIS item
**1**	**Demographics**	**PCF: 2. Demographics (age, gender, education).**
**1**	**Health**	**PCF: 1. Health (pain, fatigue, physical function)**<br>**RA-WIS** Q2 Get very stiff at work; Q4 Stress of job makes condition flare; Q6 Get good days and bad days at work; Q9 Very worried about ability to keep working; Q10 Pain or stiffness all the time at work; Q21 I get on with work but after have a lot of pain; Q22 When I'm feeling tired all the time work's a grind.
**1**	**Work History**	***PCF: 3. Economic need (income needs, quality of benefits) [added].***<br>***ECF: 9. Workplace organisation (compensation of work absences) [added].***<br>***ECF: 10 Workplace accommodations (adaptive devices; modified hours/ duties) [note added to briefly record these, as applicable, in sections 2-7].***
**2**	**Get Ready/ Travel To, From and For Work**	
	- Get ready (4 items)	**RA-WIS** Q1 Get up earlier because of condition.
	- Travel (15 items)	**WALS** Q1 Get to/from work and get to work on time.
**3**	**Workplace Access**	
	- Get Around (6 items)	**WLQ-25** Physical Demands: Q2a Walk around different work locations.<br>**WALS** Q2 Get around the workplace.<br>**RA-WIS** Q17 I have great difficulty opening some of doors at work.
	- Workplace Facilities (3 items)	

**4A**	**Completing Job Activities Physical Demands**	
	Whole section	**ECF: 7 Nature of work (physical demands).**
	- Mobility (8 items)	**WLQ-25** Physical Demands: Q2b Lift, carry, move objects >4.5kg; Q2c Sit, stand, stay in one place > 15 minutes whilst working; Q2e Bend, twist, reach while working.<br>**WALS** Q3 Sit for long periods; Q4 Stand for long periods; Q5 Lift, carry, move objects; Q7 Crouch, bend, kneel, work in awkward positions; Q8 Reach.
	- Hand Use (5 items)	**WLQ-25** Physical Demands: Q2f Use hand-held tools or equipment (e.g., phone, pen, keyboard, computer, tools).<br>**WALS** Q6 Work with hands (e.g., write, type, grasp small objects, tools, hold phone).<br>**RA-WIS** Q5 Find pressure on hands a problem.
	- Physical actions (4 items)	**WLQ-25** Physical Demands: Q2d Repeat same motions over and over whilst working.<br>RA-WIS Q11 Don’t have stamina to work like used to.
	- Computer Use (4 items)	**WLQ-25** Physical Demands: Q2f Use hand-held tools or equipment (e.g., phone, pen, keyboard, computer, tools).<br>**WALS** Q6 Work with hands (e.g., write, *type*, grasp small objects, tools, hold phone).
	- Senses (2 items)	**WLQ-25** Mental-Interpersonal Demands: Q3f Easily read or use eyes whilst working
**4B**	**Completing Job Activities: Mental; Time, Energy, Emotional Demands; Lone Worker**	

	- Time, Energy, Emotional Demands	**WLQ-25**: Time Management Demands: Q1a Work required number hours; Q1b Get going easily beginning work day; Q1c Start on job as soon as arrive at work; Q1d Do work without stopping for breaks/rests; Q1e Stick to routine or schedule.<br>**WLQ-25**: Output Demands: Q4c Finish work on time.<br>**WALS** Q9 Manage shifts or hours of work; Q10 Manage pace of work.<br>**RA-WIS** Q14 Sometimes I can't face being in work all day; Q18 I have to allow myself extra time to do some jobs.
	- Mental job demands	**ECF: 7. Nature of work (mental demands).**<br>**WLQ-25** Mental-Interpersonal Demands: Q3a Keep mind on work; Q3b Think clearly whilst working; Q3d Concentrate on work; Q3e Work without losing train of thought; Q4b Control your temper around people while working.<br>**WALS** Q12 Concentrate or keep mind on work.
	- Lone Worker	
**5**	**Relationships at Work**	
	- Supervisor	**ECF 8. Workplace Support (attitude of employer).**
	- Co-workers	**ECF 8. Workplace Support (assistance by co-workers).**<br>**ECF 9. Workplace organisation (team dynamics at work; compensation of work absences).**
	- Yours and other's reactions	**ECF 9. Workplace organisation (team dynamics at work).**
**6**	**Environmental Factors & Company Policies**	
	- Environmental Factors	

	- Sick Leave-related issues	RA-WIS Q12 I have used my holiday, so I don't have to go sick.
	- Other Company Policies	**ECF: Nature of work (job autonomy).**
**7**	**Job, Career & Home Life**	
	- Job Ability	**PCF: 4. Personal appraisal of work (career perspectives).**<br>**PCF: 5. Related skills/ abilities (work self-efficacy).**<br>**WLQ-25** Output Demands: Q5a Handle the workload; Q4b Work fast enough; Q4d Do your work without making mistakes; Q4e Feel you've done what capable of doing.<br>**WALS** Q12 Meeting current job demands.<br>**RA-WIS**: Q3 Find job about all can manage; Q7 Can get job done just slower; Q15 I have to say no to certain things at work; Q16 I've got to watch how much I do certain things at work; Q23 I'd like another job but restricted in what I can do.
	- Job Satisfaction	**PCF: 4. Personal appraisal of work (job satisfaction; career perspectives).**
	- Balance Work and Home Life	**PCF: 5. Related skills/ abilities (coping skills)** [*self-management*]<br>**PCF: 6. Work-life balance (competing social roles; quality of leisure).**<br>**ECF: 12 Non-workplace support (support from family; task assistance at home).**
	**Items not in the WES-RC:**	**ECF: 11 Economic climate/ labour regulation (income compensation; employment opportunities).**<br>**WLQ-25** Mental-Interpersonal Demands: Q3c Do work carefully; Q4a Speak with people in person, in meetings or on phone; Q4c Help other people get work done.
		**RA-WIS**: Q13 I push myself to go to work because I don't want to give in to my condition.

Work Experience Survey- Rheumatic Conditions (UK)

Key: **ECF,** Environmental Contextual factors; **PCF**, Personal Contextual Factors; **RA- WIS**, Rheumatoid Arthritis Work Instability Scale; **WALS,** Workplace Activities Limitations Scale; **WLQ-25,** Work Limitations Questionnaire – 25.
